# Interaction of Microbiota between Fish and the Environment of an In-Pond Raceway System in a Lake

**DOI:** 10.3390/microorganisms10061143

**Published:** 2022-06-01

**Authors:** Xizhao Zhang, Yang You, Fei Peng, Xuemei Tang, Yifan Zhou, Jianyu Liu, Danqing Lin, Yanfeng Zhou

**Affiliations:** 1 Key Laboratory of Freshwater Fisheries and Germplasm Resources Utilization, Ministry of Agriculture and Rural Affairs, Freshwater Fisheries Research Center, Chinese Academy of Fishery Sciences, Wuxi 214081, China; zhangxizhao@ffrc.cn (X.Z.); youyang@ffrc.cn (Y.Y.); lindq@ffrc.cn (D.L.); 2 Wuxi Fisheries College, Nanjing Agricultural University, Wuxi 214081, China; fpeng280@gmail.com (F.P.); 2020113025@stu.njau.edu.cn (X.T.); 2021113017@stu.njau.edu.cn (Y.Z.); ljy17348233012@163.com (J.L.)

**Keywords:** in-pond raceway system, large water body, microbiota, *Micropterus salmoides*, environment

## Abstract

Due to its ability to collect and remove aquaculture waste, an in-pond raceway system (IPRS) has been used to decrease the uncontrolled waste discharge in the traditional cage aquaculture method in large water bodies. However, when applied to large water bodies, its environmental performance is still lacking. This study focused on analyzing the microbial characteristics and the interaction between largemouth bass (gill and gut) microbiota and the environment (water and sediment) microbiota of an IPRS. Further, it revealed the primary relationship from the perspective of microbiota in the IPRS. The results show that (1) the alpha diversity of microbiota in the water is significantly lower than that of fish and sediment. The relationship between water microbiota and fish microbiota is limited. (2) The water microbiota inside and outside the tank showed high similarity and were not significantly affected by environmental factors. (3) The SourceTrack analysis showed that fish microbiota is one of the primary sources of sediment microbiota, and more than 15% of the sediment microbiota come from fish. Microbes such as *Faecalibacterium*, *Escherichia-Shigella*, and *Bacteroides* can significantly enrich the sediment. Our study revealed the characteristics and preliminary interaction of fish and environmental microbiota in the IPRS. It provided a reference for evaluating microbial health status in the application of IPRS in large water bodies’ aquaculture.

## 1. Introduction

Cage culture in large water bodies (rivers, lakes, reservoirs, etc.) is one of the traditional aquaculture models in China. It began to emerge in the late 1970s and then developed rapidly [1,2]. In 2019, the cage culture area reached 2.33 × 10^7^ m^2^ and produced 4.27 × 10^7^ tons of aquatic products [3]. The large-scale application of cage culture leads to the discharge of a large number of fish metabolites and uneaten residual bait into the water body, which, in turn, causes eutrophication pollution of the water body [4,5,6]. In addition, studies have shown that aquaculture activities will also affect the biodiversity of surrounding waters to a certain extent, so aquaculture activities introduced into large water bodies should be more cautious [7,8]. Exploring new production methods has become an urgent task for aquaculture to utilize large water bodies’ resources.

An in-pond raceway system (IPRS) is an efficient recirculating aquaculture system (RAS) that separates the intensive aquaculture and water purification areas. IPRS has a complete set of mechanical facilities to promote water flow, collect waste, and pump sewage [9,10,11]. IPRS has been widely used in pond culture [12,13,14] and has also been tried for large water bodies’ aquaculture. However, establishing its environmental performance and its evaluation method are the first technical questions to be answered in developing IPRS in large water bodies.

Largemouth bass (*Micropterus salmoides*) is a farmed species with important economic value; the aquaculture production of largemouth bass in China has rapidly increased from 347.3 thousand tons in 2016 to 619.5 thousand tons in 2022, with an output value of US$1.76 billion [15]. Based on the successful farming practice of largemouth bass in traditional ponds, researchers are also experimenting with the possibility of farming largemouth bass in IPRS. The growth performance, blood indicators, antioxidant status, etc. of farmed largemouth bass in IPRS have been fully understood [14,16,17,18]. In comparison, there is a lack of research on the microbial status of largemouth bass in IPRS.

The microbiota in aquaculture systems is an essential aspect of affecting the health status of farmed animals and evaluating system safety, which can reflect the health status of aquaculture systems to a certain extent [19,20]. A limited number of studies on microbiota in the IPRS have provided preliminary insights. The gut microbiota of fish cultured in IPRS is different from that of fish cultured in the pond [21]. Rapid water flow in the raceway of IPRS also affects fish gut microbiota [22]. The abundance of water microbiota in IPRS tank is affected by dissolved oxygen, stocking density, and season [23]. Benthic microbial communities in IPRS are driven by stochastic processes and may not be affected by sedimentary waste [24]. The main topic of these studies is relatively the same, that is, only one of fish microbes or environmental microbes is concerned. We lack a comprehensive study of fish microbiotas and the environmental microbiota in IPRS and an understanding of the interactions between them.

This study focused on IPRS-farmed largemouth bass in a lake. The fish and environment microbiotas in the IPRS were collected, including the gut, gill, water, and sediment. The composition and characteristics of the fish and environment in the IPRS were characterized using 16S rRNA gene high-throughput sequencing technology. We analyzed microbiota similarity in the water inside and outside the IPRS tank and explored the environmental factors that affect the microbiota. The source of sediment microbiota in the IPRS is one of the critical points of concern. We analyzed the potential source of sediment microbiota and compared significantly different microbiota between sediment and fish. Finally, we predict the functions of water microbiota and sediment microbiota.

## 2. Materials and Methods

### 2.1. Sample Collection

The IPRS was located in the lake in Fenghuang Leisure Agriculture Park, Zhangjiagang City, Suzhou City, China. It is a small, closed lake formed by artificially blocking one side of the lake bay, with an area of about 19,345.3 m^2^ and an average water depth of 4.65 m. The tank is rectangular and made of glass fiber reinforced plastic, 5-m wide, 25-m long, and 2.5-m high. The stocking density is about 33–40 kg/m^2^, and the fish are fed by commercial compound feed.

A total of 18 largemouth bass from the IPRS were collected in June 2018, and their gut and gills were removed separately. The gut and gill tissues of three fish were mixed into EP tubes as one sample, respectively. We set seven points for collecting environmental microbiota in the IPRS (Figure S1). The water samples were collected using a plexiglass water sampler, and equal amounts of water samples at 0.5 m and 1.5 m were mixed as one sample. A total of 500 mL of mixed water was filtered with 5 μm pore size and 0.45 μm pore size fiber membrane, successively, and the filter membrane was stored in an EP tube. Sediment samples were collected by grabbing an appropriate amount of surface sediments and placed in an EP tube. After collection, all samples were promptly stored in liquid nitrogen, returned to the laboratory, and stored in a −80 °C refrigerator. The operations above were performed under sterile conditions.

### 2.2. Water Environment Factors

The water samples taken at points 1–6 were used to measure water quality factors (Figure S1). Water temperature (T), pH, and dissolved oxygen (DO) were measured on-site by a HACH Hydrolab DS5 portable multi-parameter water quality analyzer; transparency (SD) was measured by a Saxon disk; and turbidity (TDS) was measured by a Hanna HI98703 portable turbidimeter. Nitrite nitrogen (NO2-N), phosphate (PO4), total dissolved nitrogen (DTN), total dissolved phosphorus (DTP), permanganate index (CODMn), ammonia nitrogen (NH3-N), total nitrogen (TN), total phosphorus (TP), and chlorophyll (Chl-a) were determined according to the “Water Quality-Technical Regulation on the Design of Sampling Programs (HJ 495-2009)” [25].

### 2.3. DNA Extraction, Amplification, and Sequencing

The tissue and environmental samples were extracted using the Neasy PowerSoil Kit (100). Standard bacterial V3-V4 region universal primers 338F (ACTCCTACGGGAGGCAGCA) and 806R (GGACTACHVGGGTWTCTAAT) were used for PCR amplification. Sequencing libraries were prepared using the TruSeq Nano DNA LT Library Prep Kit from Illumina. Before sequencing, the library was first checked on the Agilent Bioanalyzer using the Agilent High Sensitivity DNA Kit. Afterward, the library was quantified on the Promega QuantiFluor fluorescence quantitative system using the Quant-iT PicoGreen dsDNA Assay Kit. Paired-end sequencing was performed by a MiSeq 2 × 300 bp sequencer.

### 2.4. Data Analysis

First, the primers and barcodes were removed from the raw data of high-throughput sequencing, and the corresponding samples were identified and assigned. The microbiome bioinformatics analysis was performed using QIIME 2 2022.2 [26]. Raw sequence data were demultiplexed and quality filtered using the q2-demux plugin; then, denoising was performed using DADA2 [27]. All amplicon sequence variants (ASVs) were aligned to mafft [28] and used to construct a phylogeny tree using fasttree2 [29]. The alpha diversity index (observed feature, Shannon index [30], and Faith’s phylogenetic diversity [31]) and beta diversity index (Weight UniFrac [32]) were calculated after all samples were sparsed (subsampling without replacement) to 21,141 sequences per sample. SILVA 13 8.99% reference sequences [33] were assigned to ASVs using the classify-sklearn naïve Bayesian classifier [34] of the q2 feature classifier [35]. The redundancy analysis (RDA), Non-metric multidimensional scaling (NMDS), and Permutational multivariate analysis of variance (PERMANOVA) were all implemented with the Vegan v2.5-7 package [36]. The Wilcoxon test was used to compare the differences between groups in the alpha diversity index, and the Kruskal–Wallis test was used to compare the differences between sediment microbiota and fish microbiota and the differences between inside and outside tank microbiota. Variance inflation factors (VIF) were used to exclude multicollinearity factors in water factors. The significance of RDA is a permutation test performed by the anova function. A T-test was used to compare the differences of water quality factors inside and outside the tank. A traceability analysis was performed by SourceTrack2 [37] and functional prediction by FARPROTAX [38]. All visualizations in this study were implemented with R v4.0.0 (https://www.r-project.org).

## 3. Results

### 3.1. Diversity of Microbiota

**Figure 1:**
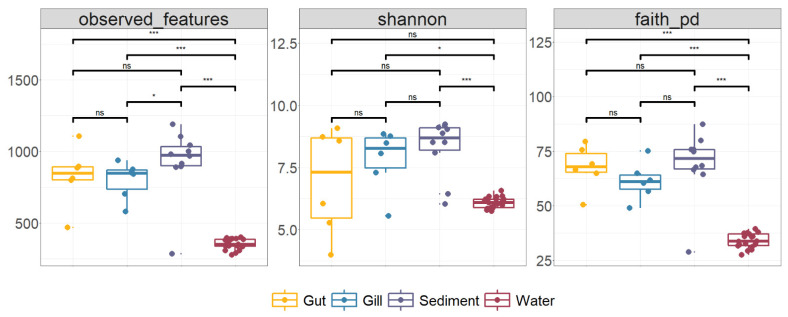
Boxplots of the alpha diversity index of the microbiota between largemouth bass and environment in the IPRS. The middle line of the box represents the median, and the upper and lower boundaries of the box represent the third quartile and the first quartile, respectively. The upper and lower whisker boundaries represent the maximum and minimum values, respectively, excluding any outliers. Dots represent values. Bracketed lines and values represent the differences between the two groups. ns, *p* > 0.5, *, *p* ≤ 0.5, ***, *p* ≤ 0.001.

The alpha diversity of fish and environment microbiota was compared. Observed features represent the number of observed ASVs, Shannon accounts for both the abundance and evenness of the taxa present. Faith pd represents the measures of biodiversity, which incorporate the phylogenetic difference between species. There are no significant differences between the gut and gill of largemouth bass (Wilcoxon, *p* > 0.05, Figure 1). Except for the significant differences in the Observed features of the sediment and gill, there was no significant difference in the other alpha diversity between sediment and fish (Wilcoxon, *p* > 0.05, Figure 1). The alpha diversity of the water was significantly lower than that of fish microbiota (Wilcoxon, *p* < 0.05, Figure 1) and only had no significant difference with the gut in the Shannon index. The alpha diversity index of sediment microbiota was significantly higher than that of water environment microbiota (Wilcoxon, *p* < 0.01, Figure 1).

**Figure 2:**
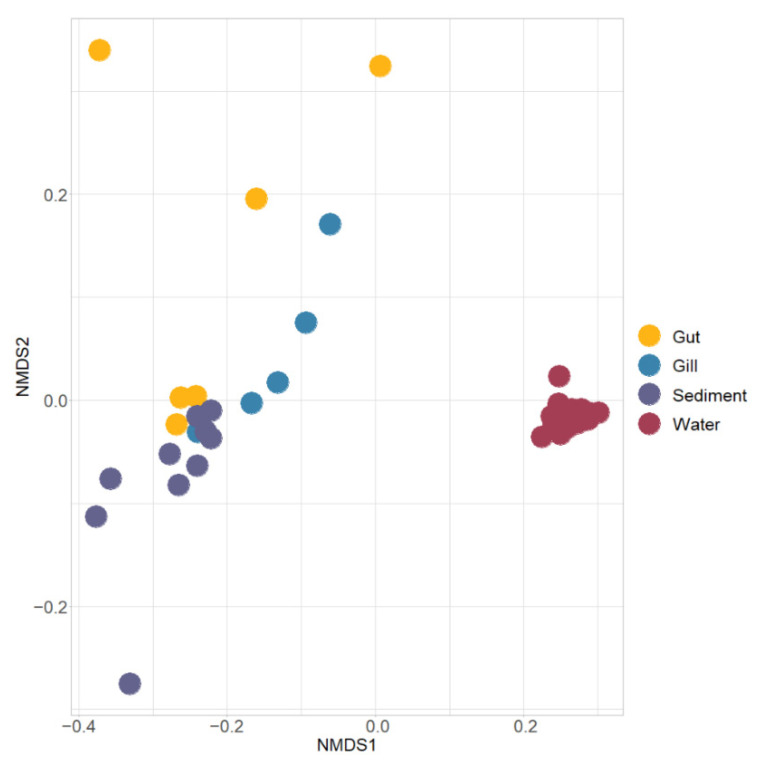
NMDS ordination based on the weight Unifrac distance between largemouth bass and the environment. The distance between the points indicates the degree of similarity between samples, and the distance indicates that the similarity was lower.

The NMDS ordination based on the weight Unifrac distance was used to compare the beta diversity among different samples in the IPRS, and PERMANOVA showed the differences between the fish and environment. The results showed that both gut microbiota and gill microbiota were significantly different from sediment microbiota and water microbiota (PERMANOVA, *p* < 0.01, Figure 2). The differences between water and fish were more significant than those between sediment and fish (Figure 2). Notably, there were no significant differences among fish microbiota (PERMANOVA, *p* > 0.05, Figure 2), while there were significant differences among environment microbiota (PERMANOVA, *p* < 0.001, Figure 2).

### 3.2. The Composition of Fish Microbiota

**Figure 3:**
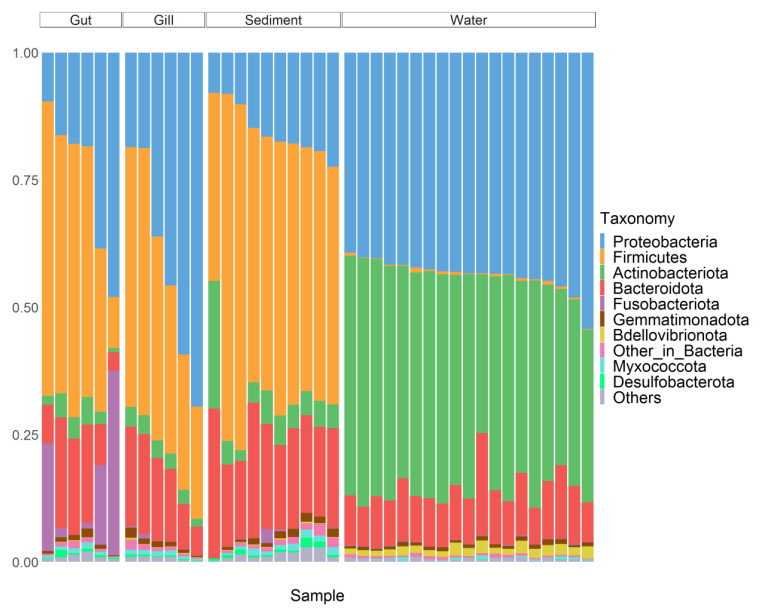
The phylum-level taxonomic composition of microbiotas in the IPRS. Only the top 10 phyla with an average proportion are displayed, and the vertical axis represents the relative proportion of each phylum.

The three phyla, Proteobacteria, Firmicutes, and Bacteroidota, constitute the main microbes in the gut and gill of largemouth bass. Fusobacteriota also accounts for a higher proportion in the gut (Mean ± Standard Error: 13 ± 5.9%, Figure 3). At the genus-level, there were some differences in the gut and gill of fish. The genus *Cetobacterium* (11 ± 5.3%) of the phylum Fusobacteriota, the genus *Plesiomonas* (13 ± 7.4%) of the phylum Proteobacteria, and the genus *Mycoplasma* (9.4 ± 6%) of the phylum Firmicutes constituted the main microbiotas in the fish gut (Figure S2). The genus *Aeromonas* (8 ± 7.3%) of phylum Proteobacteria, the inaccurately classified genus of the Lachnospiraceae family of phylum Firmicutes (5.8 ± 1.2%), and the family Rhodobacteraceae of phylum Proteobacteria (4.1 ± 1.9%, Figure S2) were the major genera in the fish gill.

### 3.3. Characteristics of Water Microbiota

The main microbes in the water were composed of Proteobacteria (44 ± 0.75%), Bacteroidota (10 ± 0.68%), and Actinobacteriota (42 ± 1.1%) at the phylum-level (Figure 3). At the genus-level, the genus *hgcI clade* (12 ± 0.59%) and *CL500-29 marine group* (11 ± 0.61%) of the phylum Actinobacteriota and the genus *Clade III* (17 ± 0.47%) of the phylum Proteobacteria constituted the main genera (Figure S2).

**Figure 4:**
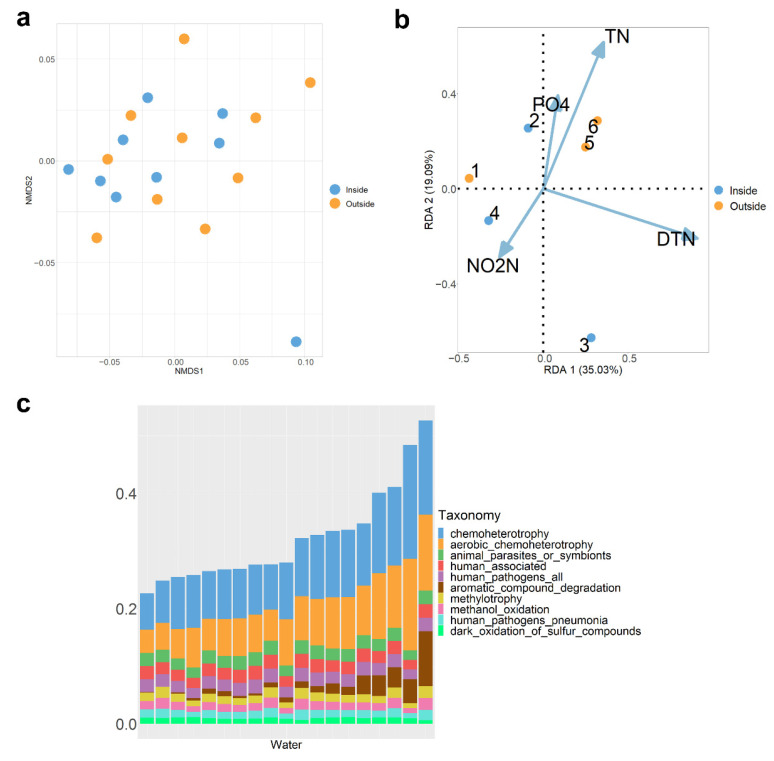
Characteristics of water microbiota. (**a**) NMDS ordination of microbiota in inside and outside water based on the weight Unifrac distance; (**b**) RDA of water microbiota and environmental factors (TN represents total nitrogen, DTN represents total dissolved nitrogen, NO2N represents nitrite nitrogen, and PO4 represents phosphate); (**c**) Top 10 predicting functions of water microbiota; the vertical axis represents the average proportion of each function.

Further analysis of the beta diversity of the water microbiota inside and outside the tank showed no significant differences (PERMANOVA, *p* > 0.05, Figure 4a). There were only 19 genera at the genus-level with significant differences between inside and outside the tank (Kruskal–Wallis Test, *p* < 0.05, Figure S3), and the proportions in the water were all less than 1%. After removing the multicollinearity factor, the four factors (TN, DTN, NO2N, and PO4) had the highest fit. The RDA results indicated that total nitrogen and total dissolved nitrogen had the highest importance for microbiota. The former factor had a positive correlation with the first axis, while the latter had a negative correlation (Figure 4b). However, the permutation test showed that the effects of environmental factors on water microbiota were insignificant (*p* > 0.05). Comparing the water quality factors inside and outside the tank, no significant difference was found (*t* test, *p* > 0.05, Table S1).

Chemoheterotrophy (11 ± 0.76%) and aerobic chemoheterotrophy (8.1 ± 0.7%) were the main functions of microbiota in water. Aromatic compound degradation (1.8 ± 0.52%), methylotrophy (1.4 ± 0.075%), and methanol oxidation (1.4 ± 0.075%, Figure 4c) were also the top 10 functions. The dark oxidation of sulfur compounds (0.98 ± 0.035%), dark sulfide oxidation (0.97 ± 0.034%), dark sulfur oxidation (0.97 ± 0.034%), and dark thiosulfate oxidation (0.97 ± 0.034%) accounted for nearly 1%.

### 3.4. Characteristics of Sediment Microbiota

**Figure 5:**
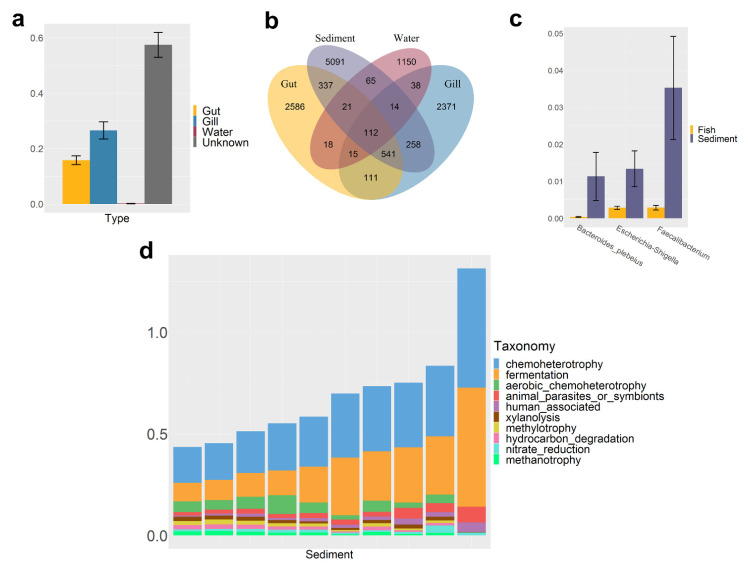
Characteristics of sediment microbiota. (**a**) SourceTrack analysis of sediment microbiota; the vertical axis represents the mean value of microbiota from potential sources, the error bars represent standard error (SE); (**b**) Venn plot of shared and unique ASVs between fish and the environment; (**c**) Significantly enriched microbiota in the sediment; the *p* value of the Kruskal–Wallis test was less than 0.05, and the average proportion in the sediment was more than 1%; the vertical axis represents the average of the three microbes, and the error bars represent the standard error (SE); (**d**) Top 10 predicting functions of sediment microbiota; the vertical axis represents the average proportion of each function.

The main phylum-level composition of sediment microbiota was Bacteroidota (20 ± 1.4%), Proteobacteria (15 ± 1.6%), and Firmicutes (52 ± 3.0%, Figure 5). At the genus-level, the inaccurately classified genus (12 ± 2.2%) of the family Lachnospiraceae of phylum Firmicutes and the genus *Bacteroides* (7.8 ± 1.7%) of phylum Bacteroidota constituted the main genera of the sediment (Figure S2).

The SourceTrack results showed that the fish microbiota was the primary source of sediment microbiota; the proportion of microbiota from the gut and gill was 16 ± 1.6% and 27 ± 3.1%, and the contribution of water microbiota to sediment microbiota was only 0.20 ± 0.067% (Figure 5a). There were 1011 shared ASVs between the fish gut and sediment, accounting for 27% of total ASVs in the gut, and 925 shared ASVs between the gill and sediment, accounting for 27% of total ASVs in the gill (Figure 5b). Three shared ASVs significantly enriched in the sediment, namely *Faecalibacterium*, *Escherichia-Shigella*, and *Bacteroides plebeius*. *Faecalibacterium* has the highest enrichment in sediment, accounting for 3.5 ± 1.4% (Kruskal–Wallis Test, *p* < 0.01); the other two ASVs were also more than 1%; *Escherichia-Shigella* was 1.1 ± 0.65%, and *Bacteroides plebeius* was 1.3 ± 0.49% (Figure 5c).

Chemoheterotrophy (29 ± 3.8%), fermentation (23 ± 4.7%), and aerobic chemoheterotrophy (4.5 ± 0.79%) were the main functions of sediment microbiota (Figure 5d). Xylanolysis (1.5 ± 0.18%), methylotrophy (1.4 ± 0.23%), hydrocarbon degradation (1.4 ± 0.22%), nitrate reduction (1.4 ± 0.26%), and methanotrophy (1.3 ± 0.21%) also exceeded 1%.

## 4. Discussion

Maintaining microbial homeostasis in fish has important implications for host health [39,40,41]. The gut and gills are the body parts of fish susceptible to bacterial infection, and the microbial status of the gut and gills under different aquaculture system deserves attention. In this study, the genera *Cetobacterium*, *Plesiomonas,* and *Mycoplasma* were the major bacteria constituting the gut microbiota of largemouth bass. Our result is highly consistent with previous studies on the gut microbiota of largemouth bass [40,42,43], indicating that the main gut microbes of largemouth bass did not change under the IPRS.

The research on the microbiota of the gill of largemouth bass is relatively lacking. This study showed that the main genera of the gill microbiota of largemouth bass include the genus *Aeromonas* of the phylum Proteobacteria and an inaccurately classified genus of the Lachnospiraceae family of the phylum Firmicutes. *Aeromonas* is a pathogen that causes red sore in largemouth bass [44], causing an inflammatory response and gut disturbances in fish [45,46,47], which can lead to a high mortality of largemouth bass in the aquaculture system. The higher abundance of *Aeromonas* in this study was mainly caused by a single sample (Figure S2), and the genus *Aeromonas* in other gill samples was maintained at extremely low levels. This result indicates the possibility of *Aeromonas* infection in largemouth bass in the IPRS, which should be paid attention to in aquaculture.

In aquaculture systems, the water environment microbiota is considered one of the main sources of the fish microbiota [39,48,49]. The shared microbes between the water and fish in the IPRS are very few compared with other aquaculture systems. The boundary between the water environment microbiota and fish microbiota in the IPRS is obvious, and the impact of the water microbiota on fish is minimal. Another difference with other aquaculture systems is that the alpha diversity of the water microbiota in this study was significantly lower than that of the fish gut and gill microbiota. The alpha diversity of water microbiota in aquaculture systems is generally significantly higher than that of fish [39,48,49]. A previous study showed that the alpha diversity of microbiota in the IPRS water was higher than that of the gastrointestinal microbes in the largemouth bass [22]. A study on RAS showed that ozone water treatment devices could lead to low diversity in the water environment [50]. However, whether the IPRS in this study can play a role in reducing the alpha diversity of water microbiota is still unclear. Because this study did not consider more factors, such as stocking time and continuous changes in the water environment, it is not yet possible to explain that the IPRS is the cause of those results. The impact of the IPRS on the interaction between water microbiota and fish microbiota deserves further exploration.

The stable water environment microbiota is vital for maintaining farmed fish’s health [51]. Studies have shown that a RAS helps maintain a stable water environment microbiota [41,52,53]. Previous studies on water microbiota in the IPRS mainly focused on the inside of the tank [22,23]. The microbial richness of the waste collection area was generally higher than that of the aquaculture area [23]. In this study, the main factors of the water environment inside and outside the tank were consistent, indicating that the water environment of the IPRS maintained relative stability. The water environment microbiota inside and outside the tank also did not show significant differences. Even at the genus-level, only 19 genera were found to be significantly different, and these genera were not predominant microbes. In addition, the water microbiota were not significantly affected by environmental factors, which also indicated the high stability of the water environment microbiota in the IPRS.

Sediment microbiota is an important part of aquaculture systems and typically exhibits high microbial diversity. The sediment microbiota in the aquaculture system is one of the primary sources of microbiota in the fish, and the fish microbiota will also settle into the sediment microbiota [39,48,49]. In this study, many fish-derived microbes were observed in the sediment, among which 16 ± 1.6% were from gut microbiota, and 27 ± 3.1% were from gill microbiota. The inaccurately classified genera of the family Lachnospiraceae of phylum Firmicutes, *Faecalibacterium*, *Bacteroides*, and *Escherichia-Shigella* were, among these shared microbes, the dominant microbes in the sediment. These bacteria occupy a certain proportion in the fish and are the most common bacteria in the intestinal tract [54,55,56,57]. In particular, the Lachnospiraceae family and the genus *Faecalibacterium* generally can produce butyrate [55,58]. The genus *Escherichia-Shigella* is found in sediments in various environments, including aquaculture sediments [59,60]. *Escherichia-Shigella* can be used as a pollutant indicator to assess the degree of pollution in the environment [61]. The genus *Escherichia-Shigella* may be considered for inclusion in the IPRS safety assessment.

Unlike traditional pond culture models, the fish in the IPRS are not in direct contact with sediments, which may limit the impact of sediment microbiota on fish. In contrast, the fish microbes may be discharged with the water flow and settle into the sediment in the IPRS. Previous studies have shown that the assembly of the sediment microbiota is not influenced by waste and is dominated by stochastic processes [24]. Our study suggests that the potential impact of fish microbiota on sediment microbiota cannot be ignored. The actual impact of fish microbiota on sediment microbiota requires further research.

The FAPROTAX function prediction focuses on predicting biogeochemical cycle processes [38]. Our results show that sediment microbiota in the IPRS is rich in functions related to carbon (fermentation) and nitrogen (such as nitrate and nitrate reduction.) cycles. In addition to participating in the carbon cycle, the water microbiota also participates in the sulfur cycle (such as dark oxidation of sulfur compounds, dark sulfide oxidation, and dark sulfur oxidation). The functional composition analysis of the environmental microbiota showed that the water and sediment microbiota play different roles in the IPRS material cycle.

## 5. Conclusions

In conclusion, we describe the microbial diversity and composition between the fish (gut and gill) and the environment (water and sediment) in the IPRS. The water microbiota in the IPRS had the lowest alpha diversity and minimal impact on fish microbiota. Comparisons at both the microbiota community- and genus-levels showed similar water microbiota inside and outside the tank. One of the important sources of sediment microbiota is fish microbiota in the IPRS. *Faecalibacterium*, *Escherichia-Shigella*, and *Bacteroides* can significantly enrich the sediment. Finally, the environment microbiota is involved in the carbon, nitrogen, and sulfur cycles in the IPRS. Our results demonstrate the characteristics and interaction of the fish and environment microbiota in the IPRS and provide a reference for the IPRS eco-environmental health assessment.

## Data Availability

The relevant scripts and codes have been uploaded to https://github.com/CesarZhang/IPRS-16S (accessed on 26 May 2022). The raw sequence reads data were submitted to NCBI under accession number PRJNA816828.

## Supplementary Materials

The following supporting information can be downloaded at https://www.mdpi.com/article/10.3390/microorganisms10061143/s1. Figure S1: Schematic diagram of IPRS sampling points; Figure S2. Genus-level taxonomic composition of microbes of the fish and environment in the IPRS; Figure S3. Different genera of bacteria in the water environment inside and outside the tank; Table S1. Water quality parameters inside and outside the IPRS.
