# Supplementary material for: Interaction of Microbiota between Fish and the Environment of an In-Pond Raceway System in a Lake
Source: Microorganisms. 2022 Jun 1;10(6):1143. doi: 10.3390/microorganisms10061143 (PMC9227127; doi:10.3390/microorganisms10061143)
Supplement: Supplementary file 1 [file microorganisms-10-01143-s001.zip › microorganisms-1738863-supplementary.pdf]

Supplementary Materials

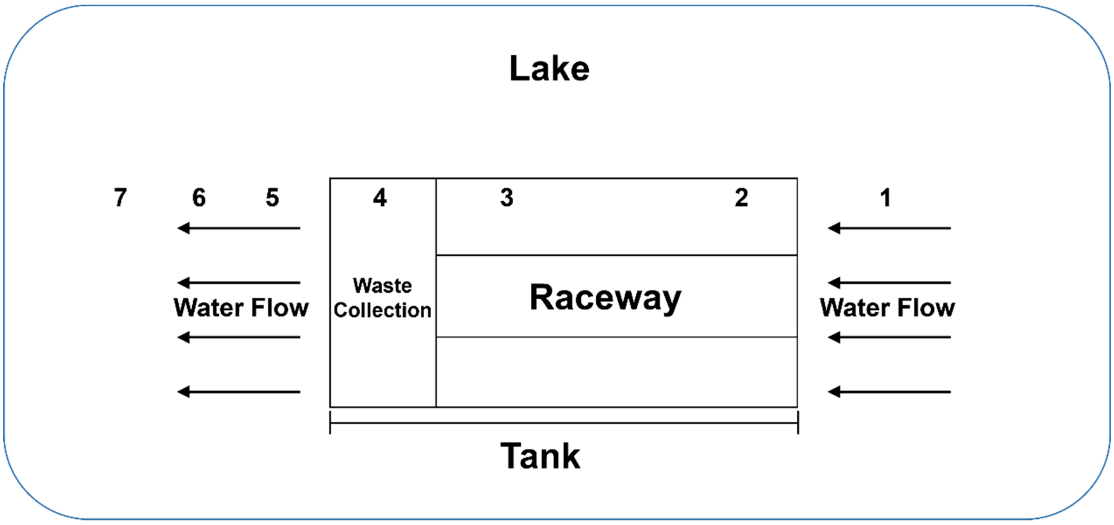

Figure S1. Schematic diagram of the IPRS sampling points. Point 6 is the zero flow rate point. Three water samples were collected at each point from 2 to 7, and one sample was collected at point 1. Three sediment samples were collected at points 5 and 6, and one sample was collected at point 1. Regularly salvage residual bait excrement at the bottom, so there is no sediment at points 2, 3, and 4.

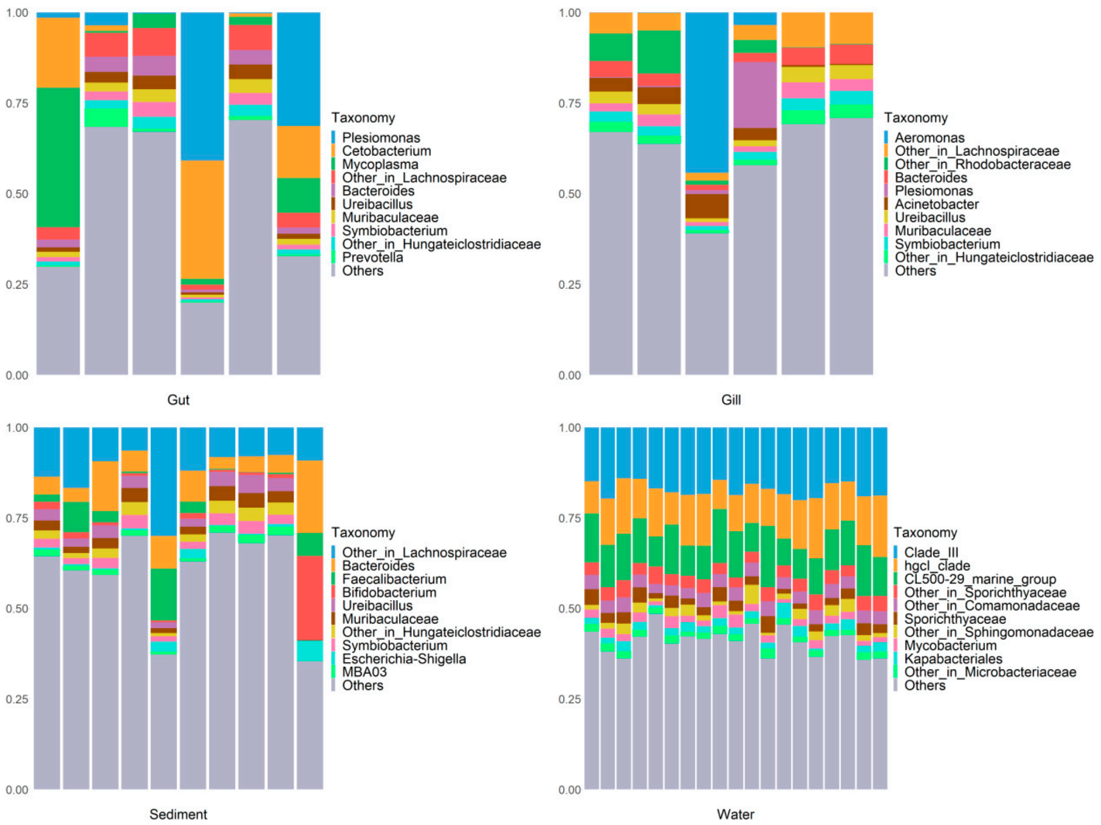

Figure S2. Genus-level taxonomic composition of microbes at the fish and environment in the IPRS. Only the top 10 genera with an average proportion are displayed, and the vertical coordinate represents the relative proportion of each genus.

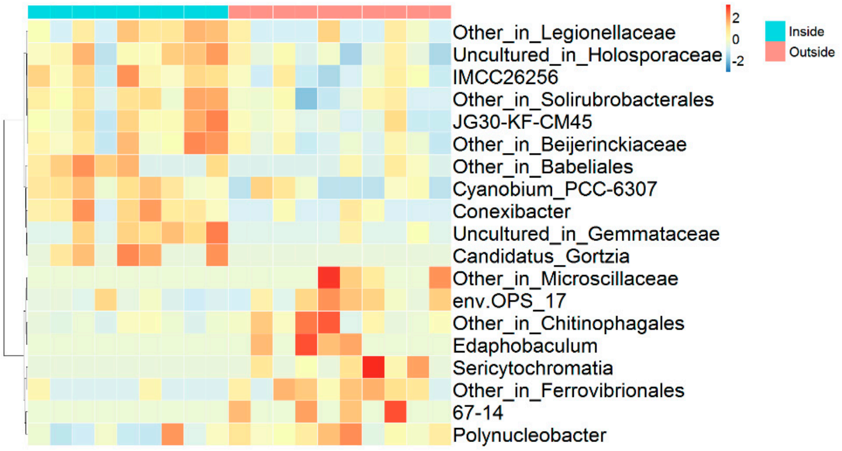

Figure S3. Different genus of bacteria in the water environment inside and outside the tank.

Table S1. Water quality parameters inside and outside the IPRS.

Site	NH3N	NO2N	PO4	TN	DTN	DTP	TP	COD MN	Chla	DO	PH	NTU	T	SD
1	0.28	0.02	0.01	4.48	3.43	0.00	0.31	5.00	36.97	12.58	7.18	22.00	27.40	31
2	1.08	0.02	0.04	5.63	3.58	0.06	0.46	7.11	36.92	6.04	7.59	31.30	24.80	22
3	0.77	0.02	0.01	4.74	4.69	0.07	0.33	7.03	52.84	6.11	7.68	34.60	24.80	21
4	0.94	0.02	0.10	5.14	4.00	0.07	0.35	7.53	55.62	6.00	7.68	32.60	24.80	22
5	1.09	0.02	0.01	5.36	4.54	0.03	0.35	7.10	61.24	6.78	7.69	29.20	25.00	20
6	0.69	0.02	0.14	5.30	4.95	0.11	0.32	6.46	26.30	7.33	7.72	26.90	25.40	21

Note: NH3N, NO2N, PO4, TN, DTN, DTP, TP, CODMN are measured in mg/L; Chla is measured in μg/L; T is measured in ℃; SD is measured in cm.
